# Local expertise anchors biodiversity documentation, but geopolitical power drives parachute discovery

**DOI:** 10.1038/s44185-026-00137-6

**Published:** 2026-05-20

**Authors:** Mario R. Moura, Raquel L. Carvalho, Karoline Ceron, Jhonny J. M. Guedes, Matheus de T. Moroti, Gabriel Nakamura

**Affiliations:** 1 Depto. de Sistemática e Ecologia, Universidade Federal da Paraíba, João Pessoa, Brazil; 2 Depto. de Zoologia, Universidade de São Paulo, São Paulo, Brazil; 3 Depto. de Biologia, Universidade Federal do Ceará, Fortaleza, Brazil; 4 Depto. de Biologia Animal, Universidade Estadual de Campinas, Campinas, Brazil; 5 Depto. de Genética, Ecologia e Evolução, Universidade Federal de Minas Gerais, Minas Gerais, Brazil

**Keywords:** Ecology, Ecology, Evolution

## Abstract

Global biodiversity documentation is limited by a persistent biodiversity knowledge split, in which high biodiverse nations have their specimens extracted and housed elsewhere. Holotypes, the name-bearing specimens required for the description of new species, are central to this imbalance. We investigated how geopolitical and socioeconomic factors shape the deposition of mammalian holotypes described over the past 35 years. While 95% of post-1990 mammal discoveries originated in the Global South, 60% of their holotypes are housed abroad, mainly in Global North institutions. Wealthier nations extracted holotype specimens disproportionately, especially from biodiverse regions with weak environmental policies, despite stronger local academic capacity improving retention. Maintaining these inequalities not only hampers efforts to reduce disparities in the distribution of type material, but also limits the discovery and revision of species. Addressing these disparities requires coordinated structural investment in local collections and taxonomic capacity, governance measures that prioritize regional deposition and equitable specimen management, and access policies that improve repatriation, mobility, and shared control over type material.

The discovery of new species typically involves comparison with type specimens of known taxa^1^, the reference material that ties scientific names to biological entities. Among these, a single specimen holds particular taxonomic significance as the name-bearing of a species—the holotype^2^. These specimens are primarily stored in museums and natural history collections, yet such institutions are unevenly distributed worldwide, with their accessibility, resources, and functionality often shaped by socioeconomic context^3,4^. Consequently, biodiversity is better documented in the economically developed Global North than in the Global South, where many species remain understudied or entirely unknown^5,6^. This imbalance may encourage researchers from wealthier nations to conduct fieldwork in biodiverse regions but deposit name-bearing specimens abroad^7^. This disconnection between local researchers and name-bearing specimens limits access to critical material, hindering efforts to efficiently document global biodiversity and achieve goals under the Post-2020 Global Biodiversity Framework^8^.

Beyond geographic and socioeconomic factors, taxonomic preferences further distort biodiversity documentation. Charismatic taxa such as mammals attract more research than less conspicuous groups like insects, fungi, or small vertebrates^9,10^. This preference has benefited mammal research with greater funding, institutional support, and international visibility^11^, fostering broader taxonomic expertise and museum infrastructure that could reduce dependence on the historical model of specimen collection and export established during the eighteenth and nineteenth centuries^12^. If these asymmetries remain evident even in mammals, they are likely to be at least as strong in less studied and less charismatic taxa. We therefore use mammals as a conservative test case for decolonial science in biodiversity documentation. Using 1116 mammal species descriptions published over the past 35 years^13^, we examined the origin and destination of name-bearing specimens and the geopolitical and socioeconomic relationships shaping their movement. We hypothesized that retention of native holotypes would increase with nation scientific capacity and strong environmental governance^12,14^, whereas appropriation of foreign holotypes would be more associated with geopolitical asymmetries, particularly in wealthier countries, with colonialist history and higher national stability^7,15^.

Despite the Global South accounting for 95% of recent mammal discoveries, nearly 60% of their holotypes are housed abroad (Fig. 1). This contrasts with Global North discoveries, where only 22% of holotypes are deposited in foreign institutions, all those in the Global North. This knowledge split between holotype housing location and resident researchers is higher than that observed for less charismatic groups, such as freshwater fishes (43% of holotypes described post-1950 and 30% of those described post-2000 are housed in foreign institutions^12^) and reptiles (50% of holotypes described post-1990^3,16^). Notably, 92.6% of specimens housed in Global South collections are native, while 92.3% of holotypes deposited in Global North institutions originate elsewhere. Because holotypes of most newly discovered species are housed abroad, researchers in the Global South may need to travel internationally to conduct taxonomic revisions^17^. When funding and logistical support for such access are limited, taxonomic revisions become less frequent in biodiversity-rich regions of the Global South^18^, reinforcing the latitudinal taxonomy gradient, where species delimitation criteria are applied less consistently in the tropics and many species remain formally unrecognized^19,20^.

**Fig. 1:**
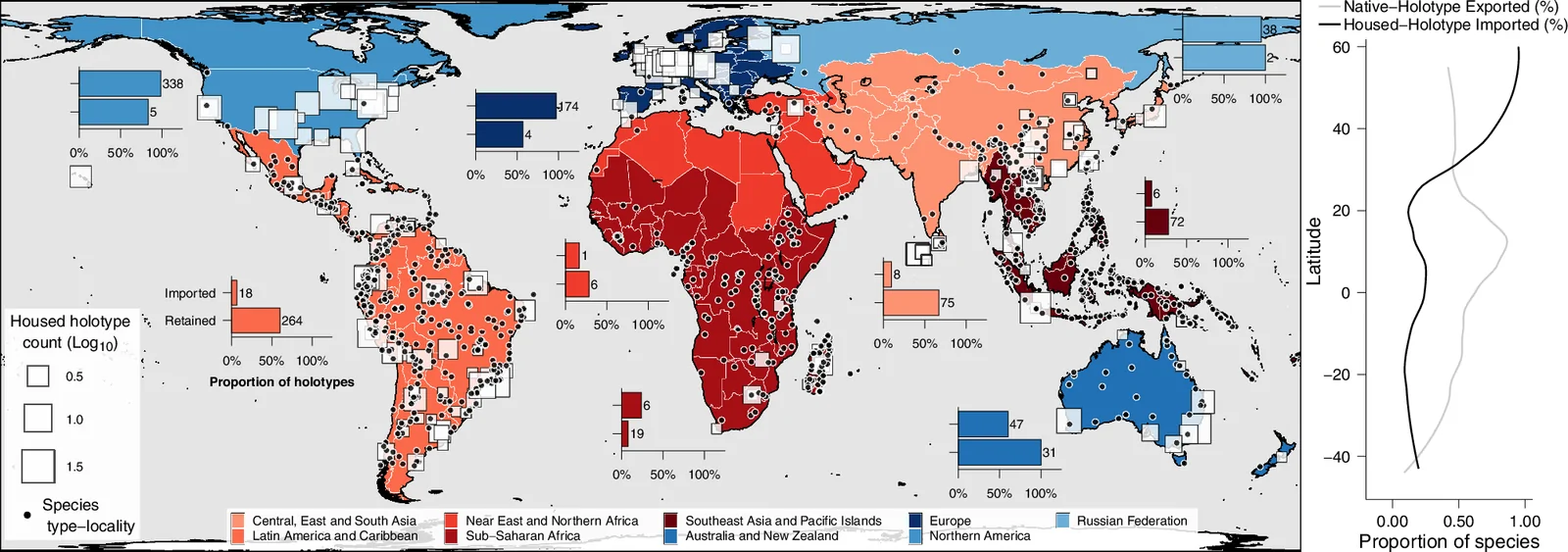
Proportion of name-bearing mammal specimens retained or imported across geopolitical regions. Black points indicate type-localities of mammal species described between 1990 and 2025, and white squares represent mastozoological collections housing the holotypes, with their size scaled to the number of holotypes per collection. Barplots show the proportion of holotypes that were retained (housed in their country of origin) or imported (housed in a foreign country). Numbers adjacent to bars are holotype counts. Reddish and bluish colors distinguish geopolitical regions associated with the Global South and Global North, respectively. The map uses an equal-area projection. See Fig. S1 for country-level spatial pattern.

These trends underscore a pronounced North–South divide in holotype retention and appropriation. Among 105 countries where new mammals were described, 68 (including 66 in the Global South) retained no holotypes. Of the 53 repository countries, only 20 housed exclusively native specimens (17 in the Global South). In contrast, 17 other countries, largely in Europe, hosted only foreign holotypes (Fig. S1). The United States held the largest collection of holotypes at 324, accounting for 28.6% of the total; 98.5% of these (319) were imported from other countries. Network analyses based on the multidirectionality and intensity of holotype flows between countries further reveal that all of the top-15 countries with most-exploited holotypes are in the Global South—especially Sub-Saharan Africa and Southeast Asia—whereas 14 of the 15 most-extractive nations are located in the Global North, with China as the only exception (Figs. 2 and S3).

**Fig. 2:**
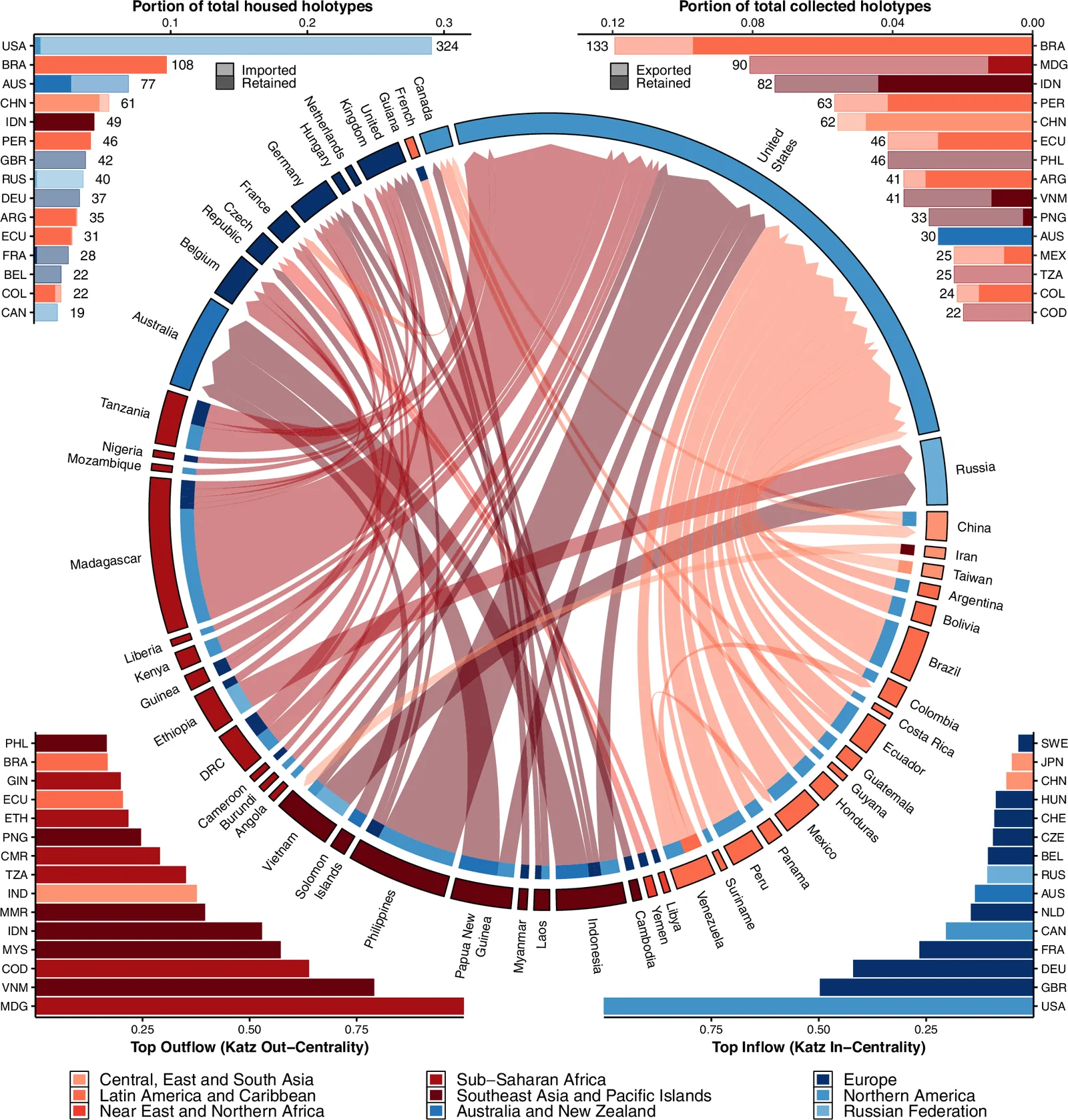
Global outflow of name-bearing mammal specimens across countries (1990–2025). Lateral barplots show top-15 countries (ISO-alpha3 code) in housing (top-left), sourcing (top-right), outflow (bottom-left), and inflow (bottom-right) of name-bearers of mammals. High inflow centrality indicates countries exerting extractive influence in parachute science networks, while high outflow centrality reflects greatly exploited countries. Only countries with at least three holotypes in foreign collections are shown. Reddish and bluish colors distinguish geopolitical regions associated with the Global South and Global North, respectively. Arrows represent source-to-repository pathways between countries (self-links omitted). See Figs. S2 and S3 for complete country-rankings and Fig. S4 for a representation of including holotype retentions.

Countries with stronger academic capacity, as reflected in a higher Human Capital Index, retain more holotypes and are less constrained by holotype extraction by different foreign institutions (outflow centrality network metric, Fig. 3). Susceptibility to these pathways of holotype extraction is amplified by weak environmental policies (proxied by low scores on the Biodiversity and Habitat Issue), a challenge that often overlaps with limited enforcement capacity in megadiverse countries^21^. Similar postcolonial shifts toward local retention of holotypes have recently been documented in plant collections, where increased national scientific capacity and stronger biodiversity governance reduced the historical export of type material to Europe and North America^14^. Historical sampling and colonial legacies further reinforce these extractive pathways (Fig. 3), with the labor of scientists and collectors in former colonies systematically exploited to export biodiversity specimens^7^. This pattern appears only weakly related to contemporary research investment, suggesting that financial resources alone do not prevent extraction when taxonomic authority and institutional decision-making remain concentrated abroad. Instead, local scientific autonomy, supported by resident expertise and leadership in species description^18,22^, may be more important than infrastructure alone for retaining holotypes near their regions of origin.

**Fig. 3:**
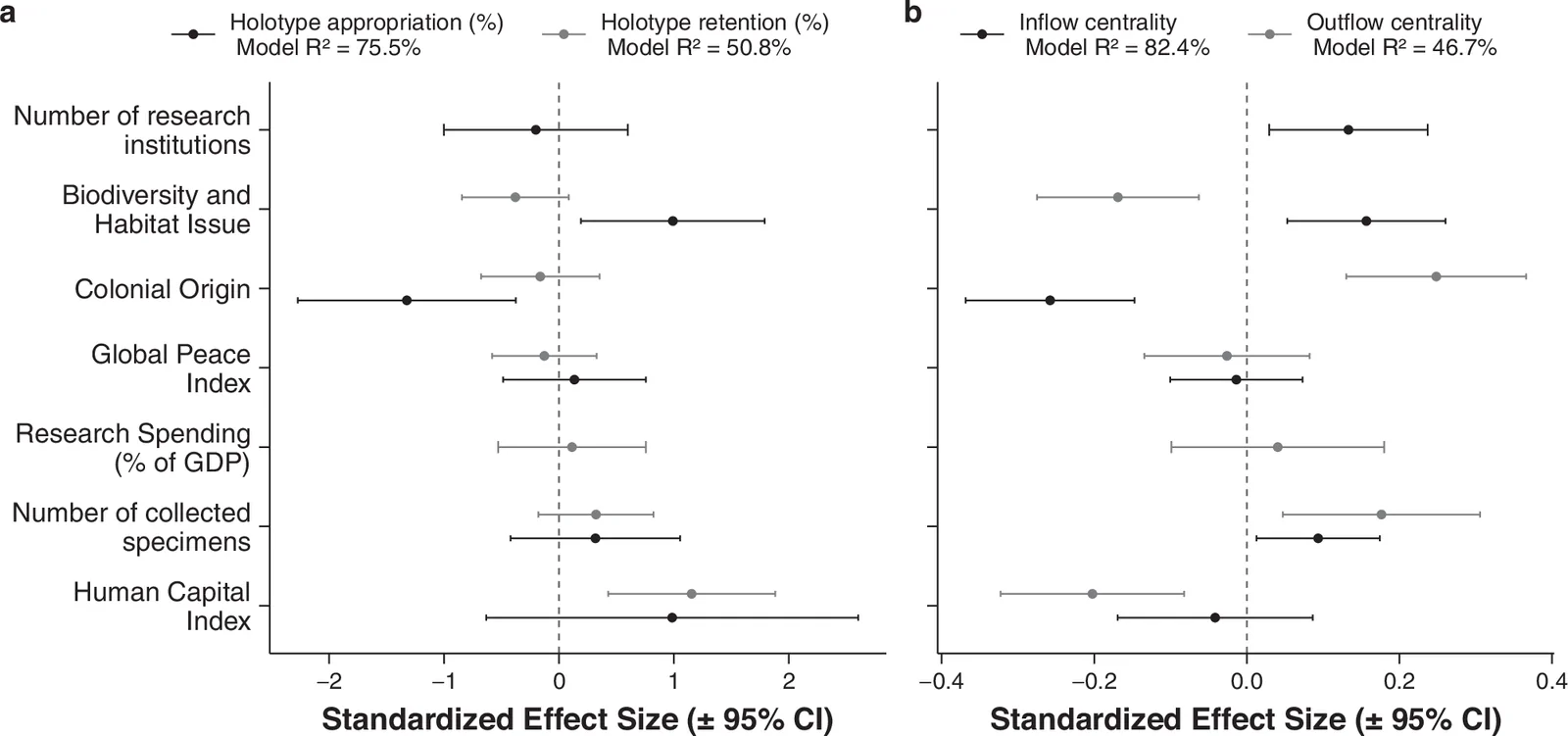
Socioeconomic drivers of global holotype dynamics. Effect sizes and 95% confidence intervals from models examining the influence of socioeconomic factors on per-country metrics. **a** shows factors affecting the retention of native holotypes and the importation of foreign holotypes. **b** shows factors affecting outflow centrality (the degree of holotype extraction by different foreign institutions) and inflow centrality (the country’s extractive influence). See Supplementary Information for coefficient estimates, standard errors, and significance values.

In contrast, both holotype appropriation and inflow (i.e., the country’s extractive influence) increase in countries with stronger conservation policies and especially in colonialist nations, indicating that destinations of mammal holotypes are driven mostly by political dominance^23^. Furthermore, holotype inflow increases with availability of research institutions and historical sampling, suggesting that established scientific infrastructure—rather than domestic academic capacity—facilitates these extractive collections. While museums with robust infrastructure may attract foreign deposits^24^, our findings reveal a pronounced power imbalance: only 3.4% of the exported mammal holotypes were described solely by domestic authors. Indeed, the vast majority (60.5%) of exported holotypes involved foreign-led research without local collaboration, underscoring a pattern of extraction where foreign scientists remove holotypes from their countries of origin. This dynamic extends beyond holotype movement itself, as taxonomic authority, revisionary work, and future species comparisons also become concentrated in foreign institutions.

Our findings reveal that while charismatic taxa attract disproportionate research attention^9,10^, this has failed to spur decolonial science practices. Mammal holotype outflow remains tied to political leverage and research infrastructure in wealthier nations. This reinforces a dependence cycle, where biodiversity-rich countries have their biodiversity mostly accessed and documented by foreign nations, while access, analytical capacity, and long-term control over name-bearing specimens vouchers remain externalized. Similar dynamics have been documented in other domains of biodiversity use, where benefits derived from biological resources are rarely retained locally. For instance, over 94% of patents based on native Brazilian plants are held by foreign entities^25^. Although international frameworks like the Convention on Biological Diversity were designed to promote fairer access and benefit-sharing, their effectiveness is limited by uneven global participation and the absence of key economies like the USA, thereby constraining their ability to counteract persistent neocolonial extraction.

Efforts to comprehensively document global biodiversity are essential but must confront the colonial structures still embedded in scientific practice^15^. We have shown that Global South nations with strong academic systems and well-established environmental policies retain more holotypes, whereas colonial history largely explains extractive pipelines. The reduction of this biodiversity knowledge split requires coordinated action across structural, governance, and access dimensions (Table 1). Structural actions include long-term investment in local collections, museum infrastructure, and taxonomic training so holotypes can remain near their regions of origin. Governance actions include low-cost policy measures such as deposition plans, regional retention of holotypes, and reciprocal co-curation agreements between institutions. Where repatriation is not immediately feasible, access measures—such as mobility funds for Global South taxonomists and strategic digitization—can reduce barriers to specimen access. Without systemic change, megadiverse nations will remain scientific marginalized, undermining global efforts to mitigate biodiversity knowledge gaps.

**Table 1:** Proposed actions to reduce the biodiversity knowledge split

Action dimension	Priority	Costs	Description
Governance	Very High	Low	**Adopt mandatory specimen deposition plans**. Journals publishing species descriptions and taxonomic revisions could require authors to submit a formal “specimen deposition plan,” justifying where type material will be stored, prioritizing institutions near the type locality to ensure local accessibility, analogous to existing data and code availability statements.
Governance	Very High	Low	**Prioritize regional over international housing**. When local housing is unavailable near the species type locality, type material should move to the nearest regional collection rather than being exported to distant Global North institutions. This improves access for local and regional researchers and reduces the need for expensive international travel or specimen loans.
Structural	Very High	High	**Strengthen local biological collections and taxonomic capacity**. Long-term investment in museums and natural history collections—from climate-controlled storage, imaging systems, and molecular infrastructure to curatorial staff, collection managers, and taxonomic training—is crucial to increase native holotype retention and local autonomy. Large grants could require a fixed proportion of funds (e.g., 20–30%) to be allocated directly to partner institutions near study sites, including infrastructure, scholarships, and technical staff support.
Access	High	Medium	**Promote repatriation of type material and mobility funds**. Physical specimens should be returned to institutions closer to their origin whenever infrastructure and legal conditions allow, restoring local access and reducing the concentration of taxonomic authority. Until repatriation is achieved, major museums and funding agencies should provide travel grants for Global South taxonomists to access collections currently held in the Global North, serving as a practical complement, not a substitute, for repatriation.
Governance + Access	High	Low-Medium	**Promote co-curation agreements**. Rather than permanent unilateral export, institutions could adopt shared custodianship models, including duplicate type series, long-term loans, reciprocal exchanges, and co-management agreements between Global North and Global South institutions. This helps retain scientific authority closer to biodiversity-rich regions.
Access	Medium	High	**Expand digitization without replacing physical access**. High-resolution digitization of holotypes and complete type series, including 2D/3D imaging, CT scans, and open-access metadata platforms, can substantially reduce barriers to taxonomic work. However, digital surrogates should complement, not replace, physical access to specimens, especially for detailed morphological revision.

Actions are classified by intervention type (structural, governance, and access), implementation priority, and relative cost for addressing scientific colonialism in biodiversity documentation.

## Methods

### Species data

We examined 1116 descriptions of mammal species published from 1990 to early 2025, as informed by Mammal Diversity Database v. 2.1^13^, an updatable and online database that provides the most current information on species-level and higher taxonomic changes of mammalian biodiversity worldwide. We focused on the 1990–2025 period because it reflects broadly similar scientific and socioeconomic conditions influencing mammal species descriptions, differing substantially from the expedition-based practices that dominated the nineteenth and early twentieth centuries. For each species in our database, we obtained information on: type-locality’s (1) latitude and (2) longitude, and the housing collection’s (3) latitude and (4) longitude. For cases where only the name of the locality was provided (e.g., names of protected areas or cities), approximate coordinates were sought using gazetteers or tools like Google Earth (https://earth.google.com). Two species, the Northern Talapoin Monkey (*Miopithecus ogouensis*) and the Giant Muntjac (*Muntiacus vuquangensis*), were excluded from the dataset due to missing information on either their type-locality or housing collection.

### Country data

Using coordinates of type-localities and housing collections of name-bearer mammals, we extracted the geopolitical classification adopted by the United Nations^26^ to obtain the following: (1) the ISO-alpha3 code, a three-character abbreviation representing each country’s name; (2) the geopolitical location of the country in the Global North or Global South; and (3) the geopolitical region to which the country belongs (Sub-Saharan Africa; Latin America and the Caribbean; North America; Australia and New Zealand; Central, Eastern, and Southern Asia; Europe; Near East and Northern Africa; Southeast Asia and Pacific Islands). Japan, South Korea, Israel, and French Guiana are classified as part of the Global North, although they are typically included in geopolitical regions dominated by countries in the Global South. For greater geographic detail, we removed the Russian Federation from Europe to represent it as an independent geopolitical region. All operations were conducted in R v. 4.5.0^27^.

To test how local scientific capacity, governance, geopolitical asymmetries, and historical extraction shape holotype retention and appropriation, we computed 8 country-level socioeconomic and research effort variables. First, we extracted (1) the number of biodiversity institutions (i.e., museums, herbaria, and universities) based on the dataset available in the R package *CoordinateCleaner*^28^. We used the Open Alex database (https://api.openalex.org/) to retrieve the number of available biodiversity institutions for 11 countries lacking data on the biodiversity institutions counts (Antigua and Barbuda, Bonaire, Republic of the Congo, Grenada, Kyrgyzstan, Mali, Eswatini, Samoa, Seychelles, Saint Vincent and the Grenadines, and Timor-Leste). Next, we extracted the (2) number of preserved specimens collected in each country from the Global Biodiversity Information Facility (GBIF), downloading all available mammal records with coordinates^29^, yielding 3,366,092 records after removing duplicates. Using information on the locality of housing collections, we also computed the number of preserved specimens housed in each country, but we refrained from using it in the analysis due to its high data missingness.

We used the *wb_data* function from the *wbstats* R package^30^ to retrieve World Bank Open Data indicators on: (3) percentage of GDP Expenditure in Research and Development. Since this indicator is provided annually in the World Bank Open Data platform, we averaged their respective values between 1990 and 2025. Importantly, socioeconomic data is not free from geopolitical biases, and Global South nations present missing data more frequently than those in the Global North. For countries with missing data in one or two socioeconomic factors, we imputed values using relevant regional averages. Specifically, we applied the Least Developed Countries (LDC) average of percentage of GDP Expenditure in Research and Development to 11 nations (Benin, Cameroon, Central African Republic, Eswatini, Guinea, Liberia, Malawi, Samoa, São Tomé and Príncipe, Timor-Leste) and the Latin America and Caribbean average to five non-LDC countries (Antigua and Barbuda, Barbados, Grenada, Guyana, and Suriname).

We sourced additional country-level indicators from the Quality of Government (QoG) database^31^, including: (4) biodiversity and habitat issue category, measures national efforts to protect ecosystems and prevent species loss; (5) Global Peace Index, assesses national peacefulness based on conflict, safety, and militarization; (6) Human Capital Index, evaluates how well countries develop education and health for productivity; (7) Human Development Index, summarizes achievements in health, education, and living standards; and (8) colonial origin, indicates a country’s historical colonization background. The latter variable was binarized (0 = no colonial origin, 1 = colonial origin). Each country’s value was averaged across the available QoG time-series from 1990 to 2025. The Global Peace Index for seven countries was imputed using regional averages, including three LDC nations (Samoa, Sao Tome and Principe, and Solomon Islands) and for Seychelles (acknowledging that our data collection preceded its 2014 graduation from the LDC category), while the Latin America and the Caribbean average was used for Antigua and Barbuda, Suriname, and Saint Vincent and the Grenadines.

### Metrics of retention and appropriation of name-bearers

To avoid biases from country area effects, we calculated the proportion of native name-bearing specimens retained per country, relative to the total number of native mammal name-bearing specimens discovered in each country. As a metric of name-bearing specimen appropriation, we computed the proportion of imported name-bearing specimens relative to the total housed in each country.

### Metrics of extractive flows in holotype networks

We constructed a bipartite network to analyze the holotypes’ global flows between countries based on their type localities and the housing collections of name-bearing mammals. Weights represented the number of occurrences between country pairs. At the node level, we computed Katz centrality to analyze the influence of a node (i.e., country) within a network, based on its number of immediate neighbors and the direct and indirect paths to other countries in the network^32^. The attenuation factor (α) was set to 0.1, penalizing contributions from more distant connections (range: 0–0.2). This calculation was performed using the *katzcent* function from the *centiserve* R package^33^. Preliminary inspections showed no significant area effect on inflow and outflow metrics.

### Data analysis

Some predictor variables (number of institutions, number of collected specimens, and GDP expenditure in research and development) vary over orders of magnitude and were log_10_-transformed to reduce skewness. Due to a lack of socioeconomic data for some countries, the analyses of holotype retention and outflow were based on 52 nations with at least three post-1990 mammal discoveries in their territory. These selected countries included the sourcing localities for 92% of all holotypes, and housed 97.3% of holotypes that remained in their country of origin. Similarly, the assessment of holotype appropriation and inflow included 32 nations that housed at least three holotypes, accounting for 97.6% of all housing localities, and 98% of all imported holotypes. All predictors were centered and standardized to allow comparability of model coefficients.

We examined multicollinearity in predictor variables in each dataset separately (52-nations and 32-nations datasets), using the Variance Inflation Factor (VIF) computed via the *usdm* package^34^. Predictors with VIF values > 5 were considered to exhibit strong multicollinearity and were removed from the models^35^ (Table S1). For both country-level datasets, analyses included the following predictors: Global Peace Index, Human Capital Index, colonial origin registered as binary, Biodiversity and Habitat Issue, number of collected specimens. For the 53-nation dataset, which modeled holotype retention and outflow as response variables, we also included the percentage of GDP expenditure on research and development. For the 32-nation dataset, which modeled holotype appropriation and inflow, we additionally included the number of biodiversity institutions. The final predictor sets were selected on a per-dataset basis to minimize multicollinearity, and the variables differing between datasets (i.e., number of biodiversity institutions and percentage of GDP expenditure on research and development) are strongly correlated (r = 0.39, *p* < 0.001).

To evaluate how scientific capacity, governance, and geopolitical factors influence holotype dynamics, we built four generalized linear models, one for each response variable (success in the retention of holotypes, success in the importation of foreign holotypes, and two network variables of inflow and outflow centrality for holotype transfers). Success in the retention/importation of holotypes was modeled with a beta-binomial error distribution, whereas holotype outflow/inflow centrality were modeled with a gamma error. All models were built using the *glmmTMB* package^36^. Pseudo-R² were calculated with the *performance* package^37^. Tjur’s method was used to compute pseudo-R² for Beta-binomial models, whereas Nagelkerke’s method was used to obtain R² for models with Gamma error distribution. Model diagnostics were performed using the *DHARMa* package^38^ (see Figs. S6−S9 and Data availability^39^).

## Supplementary information

44185_2026_137_MOESM1_ESM

## Data Availability

The data to reproduce the results of this study are available via Zenodo Digital Repository^39^.

## Code availability

The code for reproducing the results of this study is available at Zenodo Digital Repository^39^.
