# Supplementary material for: Local expertise anchors biodiversity documentation, but geopolitical power drives parachute discovery
Source: NPJ Biodivers. 2026 May 20;5:29. doi: 10.1038/s44185-026-00137-6 (PMC13627701; doi:10.1038/s44185-026-00137-6)
Supplement: Supplementary file 1 [file 44185_2026_137_MOESM1_ESM.pdf]

SUPPLEMENTARY INFORMATION

Local expertise anchors biodiversity documentation, but geopolitical power drives parachute discovery

Mario R. Moura[1*], Raquel L. Carvalho[2], Karoline Ceron[3], Jhonny J. M. Guedes[4], Matheus de T. Moroti[4], Gabriel Nakamura[5]

Correspondence to: mariormoura@gmail.com

Full text DOI.: 10.1038/s44185-026-00137-6

This PDF file includes

Supplementary Tables

Supplementary Figures

SUPPLEMENTARY TABLES

Supplementary Table 1.

Variation Inflation Factor (VIF) for socioeconomic variables per country. Analyses of holotype retention and outflow centrality used the 52-nation dataset, while analyses of holotype appropriation and inflow centrality used the 32-nation dataset.

Variable	52-nations dataset	32-nations dataset
Biodiversity and Habitat Issue	1.059	2.043
Colonial Origin	1.356	2.244
Global Peace Index	1.140	1.475
Human Capital Index	1.488	3.270
Research Spending (% of GDP)	1.771	-
Number of collected specimens	1.301	1.366
Number of research institutions	-	2.080

Supplementary Table 2.

Results of the beta-binomial generalized linear model (GLM) examining factors associated with holotype retention. The table presents coefficient estimates (log-odds scale), standard errors, and significance values. Significant predictors at the $p < 0.05$ level are indicated in bold face. Total number of observations = 52. Model R^2 = 50.8%.

Coefficients	*Estimates*	*CI*	*P-value*
Intercept	-1.34	-1.84 – -0.85	**<0.001**
Research Spending (% of GDP)	0.11	-0.53 – 0.76	0.731
Biodiversity and Habitat Issue	0.32	-0.18 – 0.82	0.208
Global Peace Index	-0.38	-0.85 – 0.09	0.110
Human Capital Index	-0.13	-0.58 – 0.33	0.581
Colonial Origin	1.15	0.43 – 1.88	**0.002**
Number of collected specimens	-0.16	-0.68 – 0.35	0.534

Supplementary Table 3.

Results of the beta-binomial generalized linear model (GLM) examining factors associated with holotype appropriation. The table presents coefficient estimates (log-odds scale), standard errors, and significance values. Significant predictors at the $p < 0.05$ level are indicated in bold face. Total number of observations = 32. Model R^2 = 75.5%.

Coefficients	*Estimates*	*CI*	*P-value*
Intercept	-0.55	-1.27 – 0.18	0.138
Number of institution	-0.20	-1.00 – 0.60	0.621
Number of collected specimens	0.32	-0.42 – 1.05	0.400
Biodiversity and Habitat Issue	0.99	0.19 – 1.79	**0.015**
Global Peace Index	0.13	-0.49 – 0.76	0.673
Human Capital Index	0.98	-0.63 – 2.60	0.233
Colonial Origin	-1.32	-2.27 – -0.38	**0.006**

Supplementary Table 4.

Results of the Gamma generalized linear model (GLM) examining factors associated with holotype outflow centrality. The table presents coefficient estimates (log-scale), standard errors, and significance values. Significant predictors at the $p < 0.05$ level are indicated in bold face. Total number of observations = 52. R^2 Nagelkerke = 46.7%.

Coefficients	***Estimates***	***CI***	***P-value***
Intercept	1.37	1.27 – 1.47	**<0.001**
Research Spending (% of GDP)	0.04	-0.10 – 0.18	0.568
Number of collected specimens	0.18	0.05 – 0.31	**0.007**
Biodiversity and Habitat Issue	-0.17	-0.27 – -0.06	**0.002**
Global Peace Index	-0.03	-0.13 – 0.08	0.638
Human Capital Index	-0.20	-0.32 – -0.08	**0.001**
Colonial Origin	0.25	0.13 – 0.37	**<0.001**

Supplementary Table 5.

Results of the Gamma generalized linear model (GLM) examining factors associated with holotype inflow centrality. The table presents coefficient estimates (log-scale), standard errors, and significance values. Significant predictors at the $p < 0.05$ level are indicated in bold face. Total number of observations = 32. R^2 Nagelkerke = 82.4%.

Coefficients	***Estimates***	***CI***	***P-value***
Intercept	0.50	0.43 – 0.57	**<0.001**
Number of research institutions	0.13	0.03 – 0.24	**0.012**
Number of collected specimens	0.09	0.01 – 0.17	**0.024**
Biodiversity and Habitat Issue	0.16	0.05 – 0.26	**0.003**
Global Peace Index	-0.01	-0.10 – 0.07	0.756
Human Capital Index	-0.04	-0.17 – 0.09	0.525
Colonial Origin	-0.26	-0.37 – -0.15	**<0.001**

SUPPLEMENTARY FIGURES

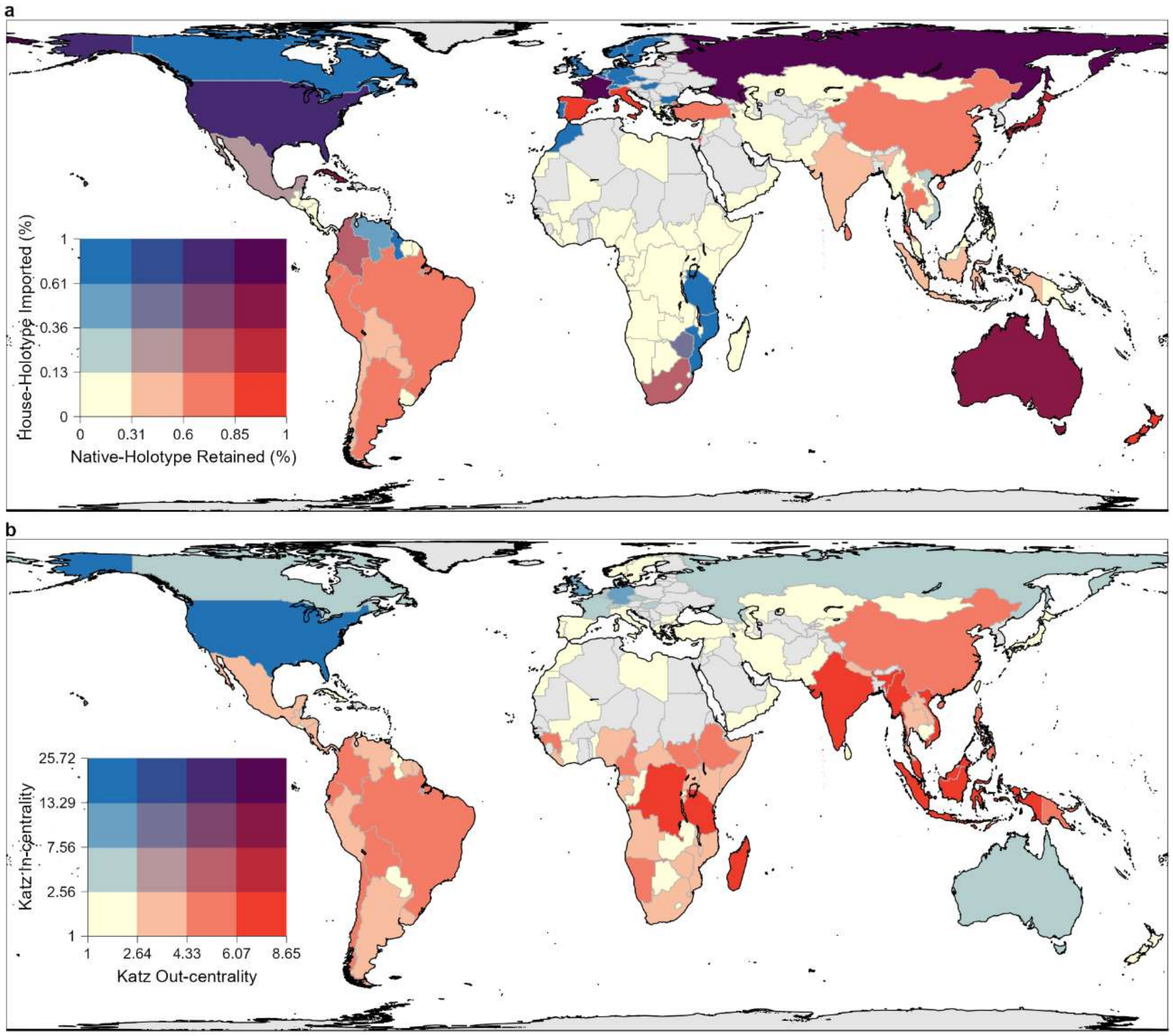

Supplementary Figure 1.

Geographical patterns of name-bearing mammal specimens transfer across countries. a Per country proportion of housed holotypes imported and native holotypes exported. **b** Holotype flows across countries as indicated by Katz In-centrality and Katz Out-centrality. Colour scales in the bivariate choropleth represent Jenks breaks intervals for each variable, as indicated in the inset plot. Countries lacking values in one network dimension due to unilateral sourcing or housing dynamics were assigned the minimum observed value for the missing axis exclusively for visualization purposes, preserving representation within the continuous bivariate colour scale. Maps draw using an equal area projection.

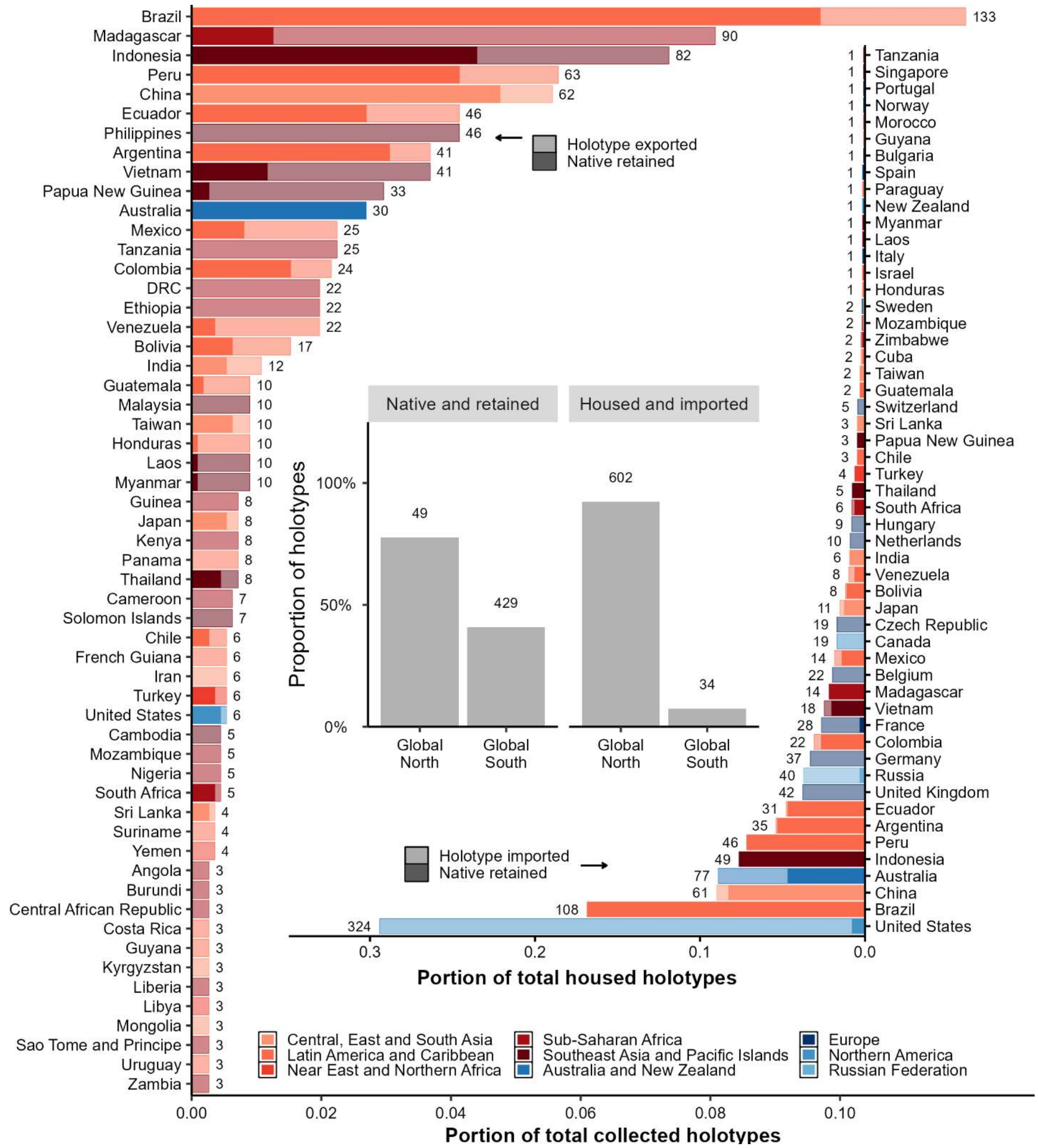

Supplementary Figure 2.

Per-country sourcing and housing counts of name-bearing mammal specimens (1990–2025). Barplots show the proportions of holotypes retained locally versus exported (sourcing) and imported versus natively retained (housing). Numbers adjacent to bars indicate absolute values. Only countries with ≥3 described species are included.

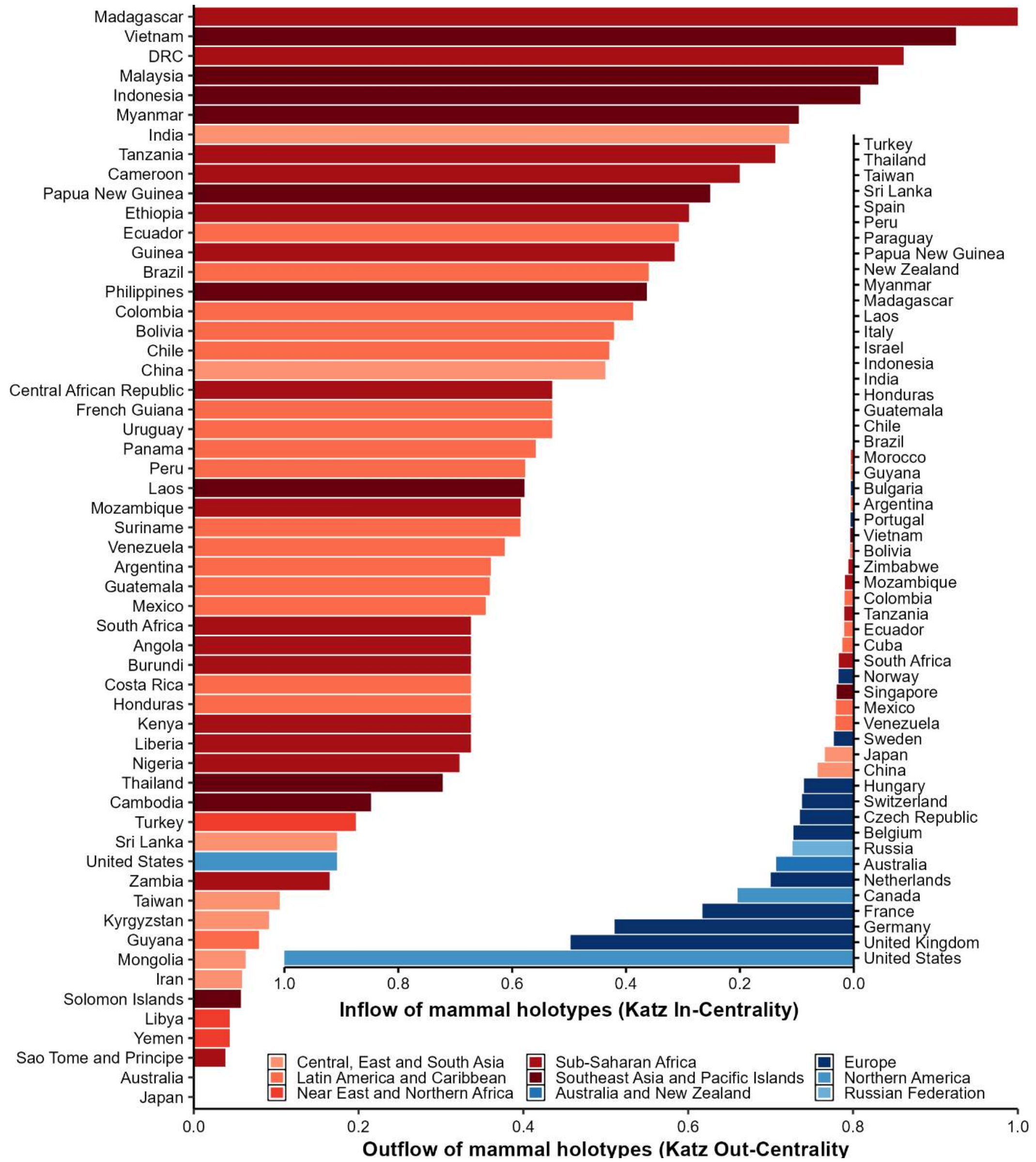

Supplementary Figure 3.

Per-country outflow and inflow centrality of name-bearing mammal specimens (1990–2025). Katz centrality metrics were rescaled to a range of 0–1. For outflow centrality, only countries with ≥3 described species are shown. High inflow centrality indicates countries exerting extractive influence, while high outflow centrality reflects exploited countries.

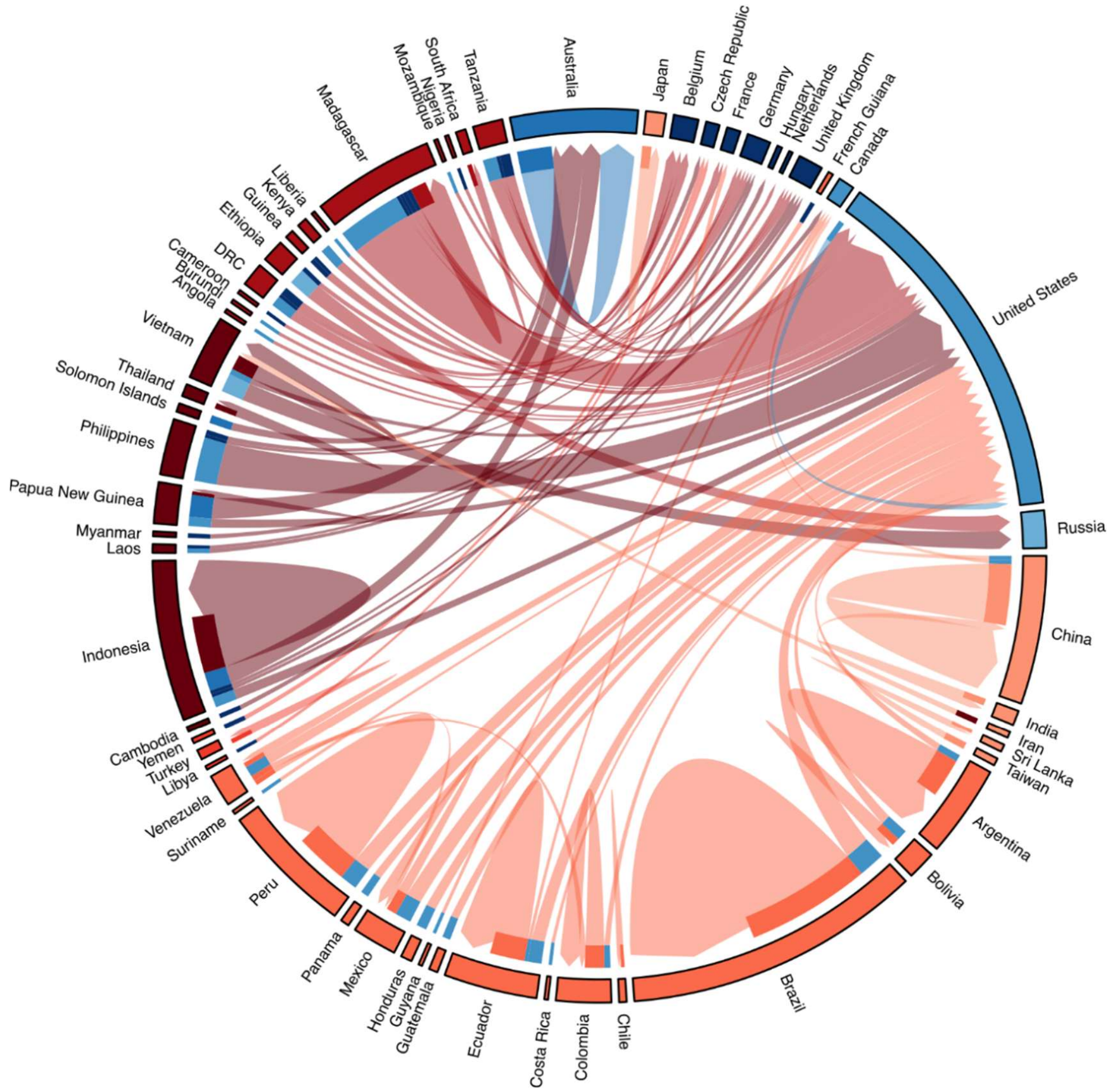

Supplementary Figure 4.

Global flow of name-bearing mammal specimens across countries (1990-2025). Only countries with at least three holotypes housed in foreign collections are shown (colors match Fig. 1). Arrows indicate source-to-repository pathways, with origin countries pointing to host nations.

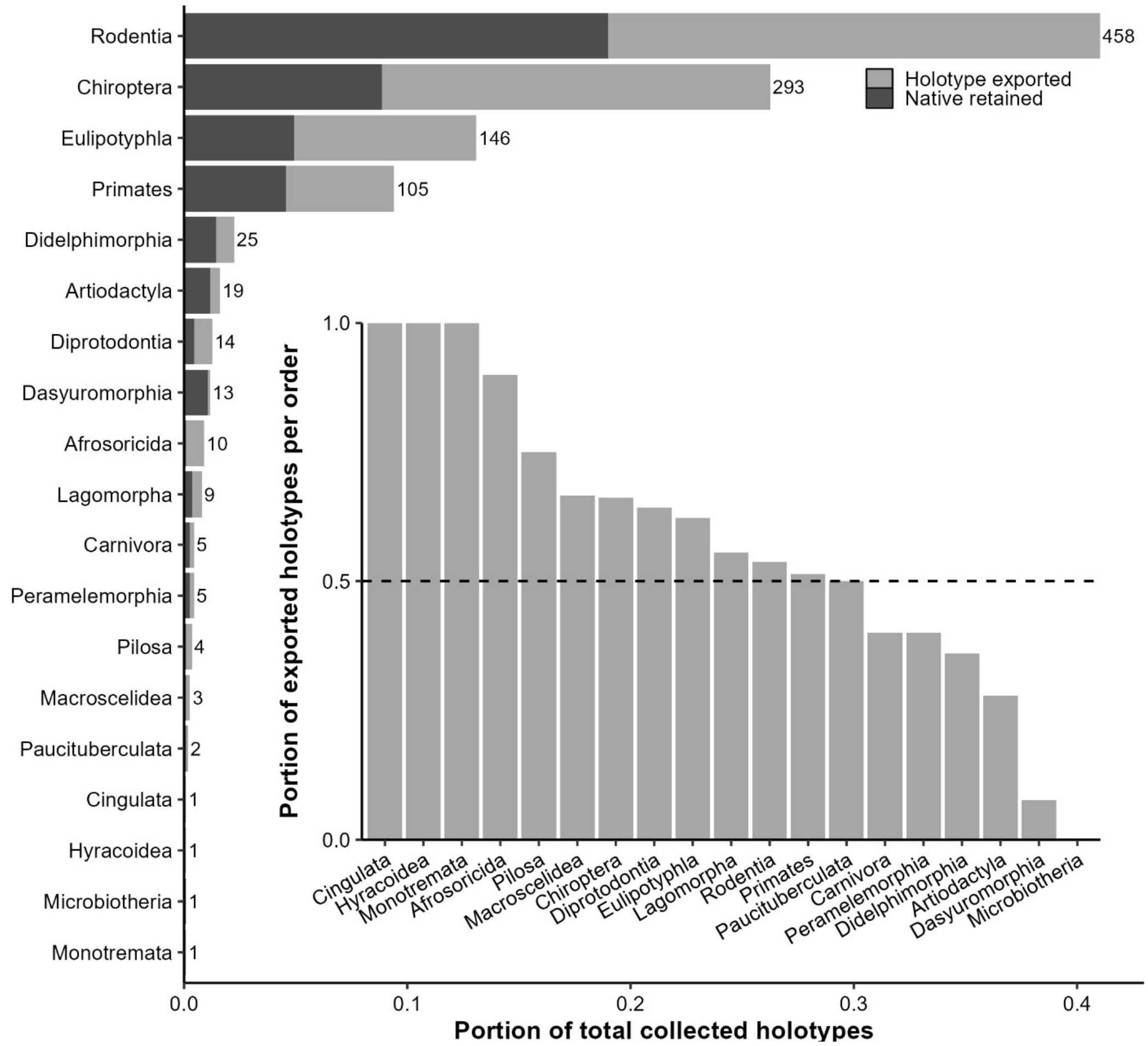

Supplementary Figure 5.

Per-order portion of retained and exported name-bearing mammal specimens (1990–2025). Barplots show the proportions of holotypes retained locally versus exported to foreign institutions. Numbers adjacent to horizontal bars indicate holotype counts per order.

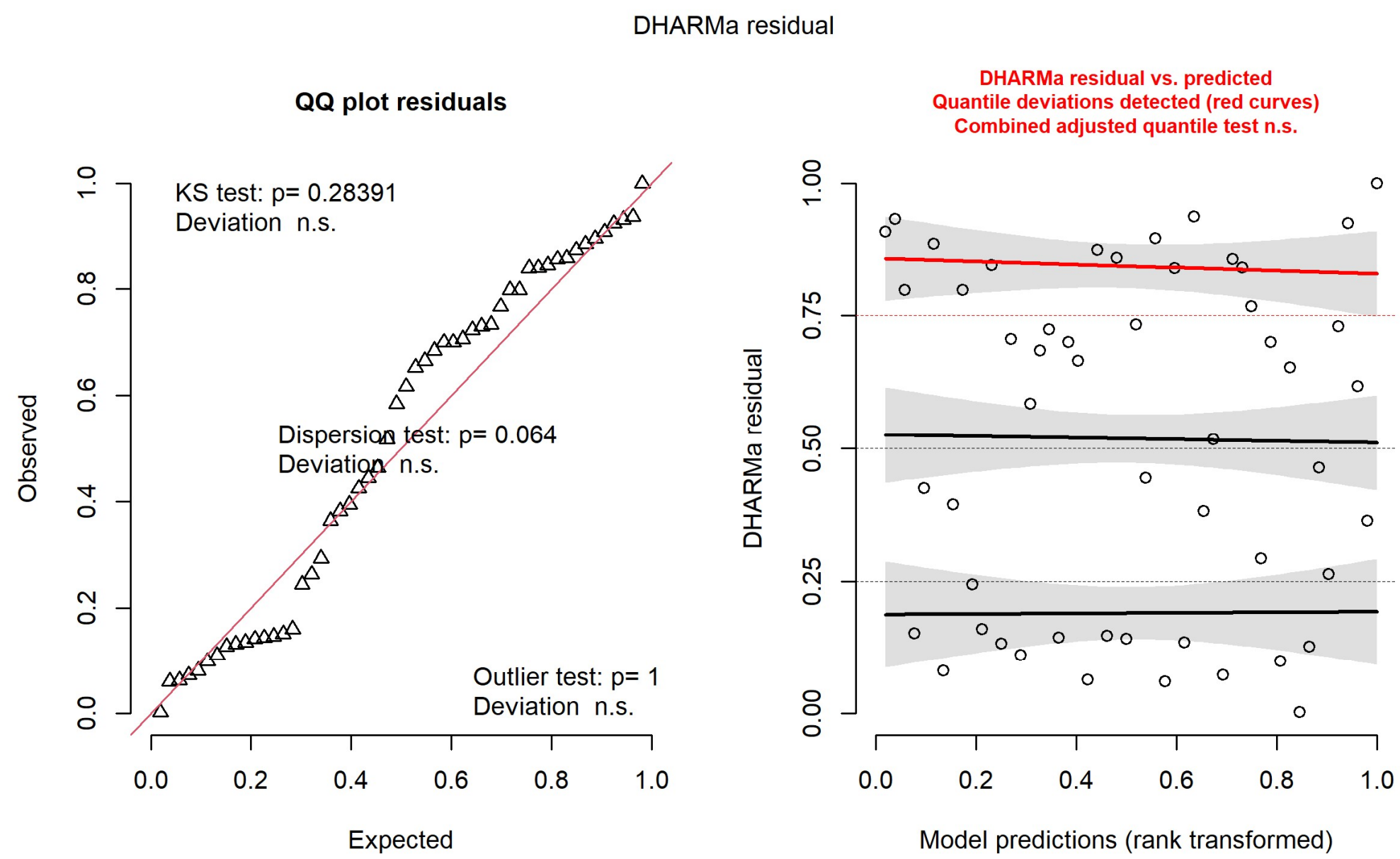

Supplementary Figure 6.

DHARMa residual diagnostics for the model of holotype retention. (a) Q-Q plot of scaled residuals against uniform distribution. (b) Residuals plotted against model predictions. A significant deviation in one diagnostic test does not necessarily invalidate our results when considered alongside others. The QQ plots indicated a generally good fit, and residual plots showed only minor deviations in one or two quantiles. This is not a major concern, particularly given our reasonably sample size.

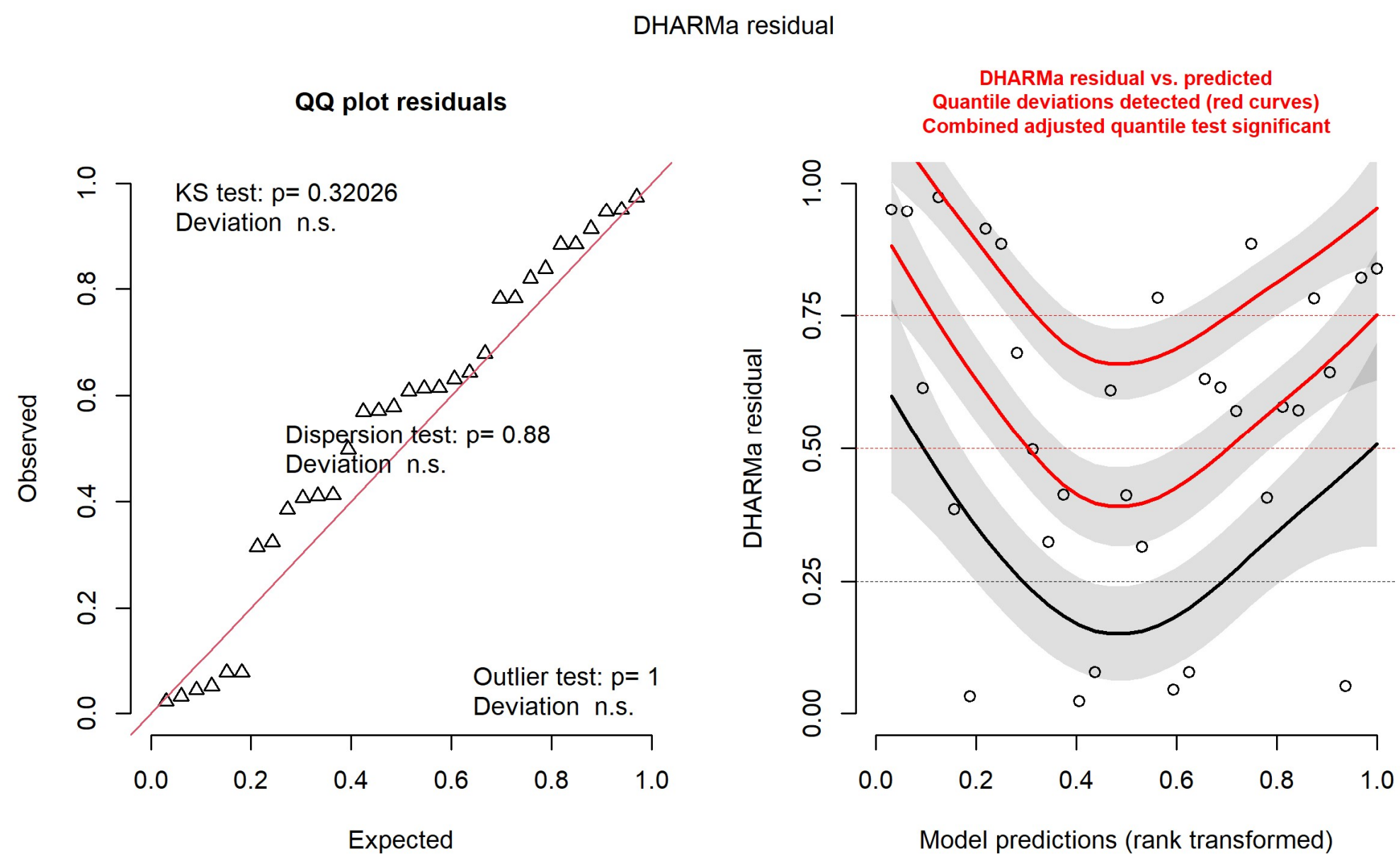

Supplementary Figure 7.

DHARMa residual diagnostics for the model of holotype appropriation. (a) Q-Q plot of scaled residuals against uniform distribution. (b) Residuals plotted against model predictions. A significant deviation in one diagnostic test does not necessarily invalidate our results when considered alongside others. The QQ plots indicated a generally good fit, and residual plots showed only minor deviations in one or two quantiles. This is not a major concern, particularly given our reasonably sample size.

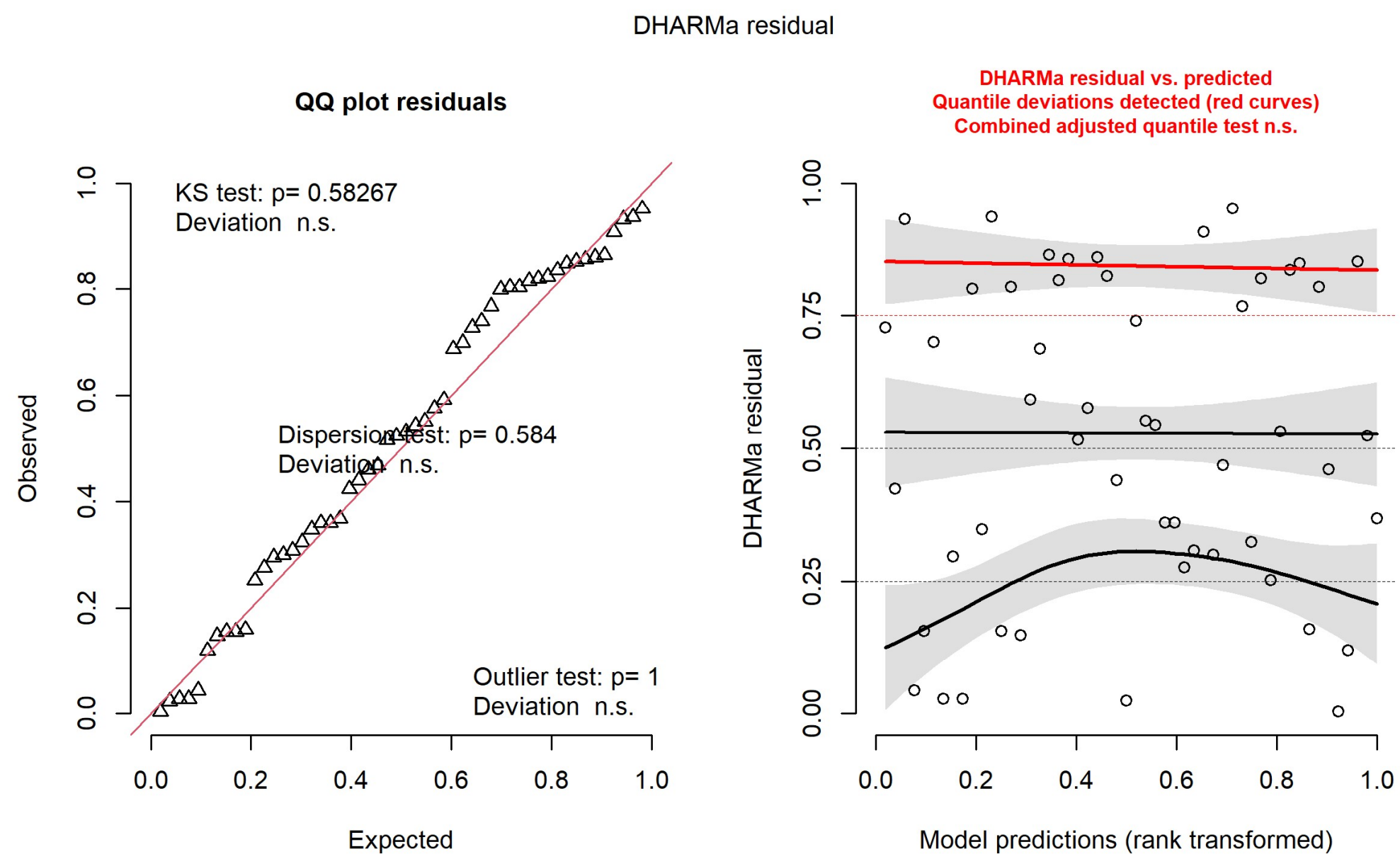

Supplementary Figure 8.

DHARMa residual diagnostics for the model of holotype outflow centrality. (a) Q-Q plot of scaled residuals against uniform distribution. (b) Residuals plotted against model predictions. A significant deviation in one diagnostic test does not necessarily invalidate our results when considered alongside others. The QQ plots indicated a generally good fit, and residual plots showed only minor deviations in one or two quantiles. This is not a major concern, particularly given our reasonably sample size.

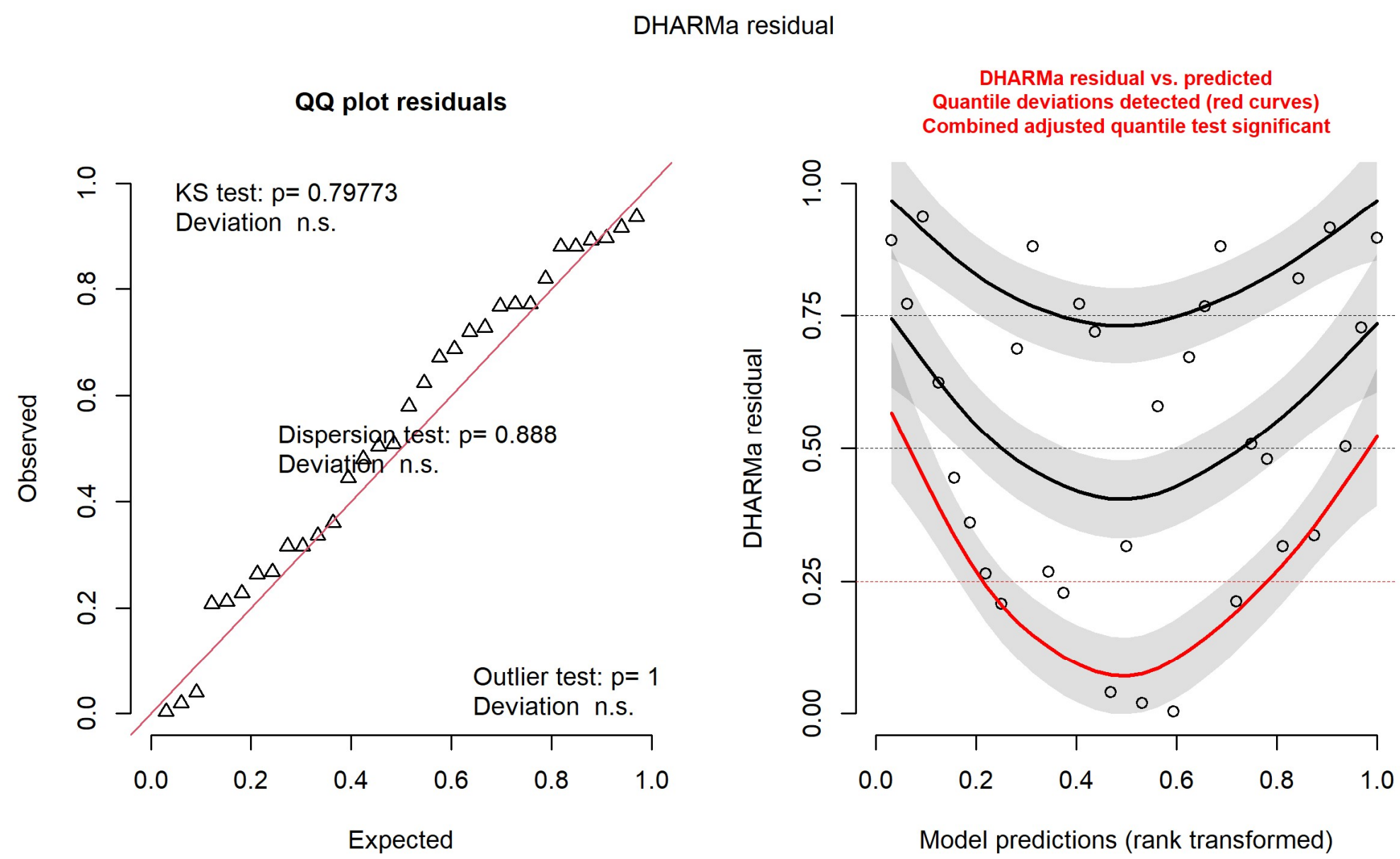

Supplementary Figure 9.

DHARMa residual diagnostics for the model of holotype inflow centrality. (a) Q-Q plot of scaled residuals against uniform distribution. (b) Residuals plotted against model predictions. A significant deviation in one diagnostic test does not necessarily invalidate our results when considered alongside others. The QQ plots indicated a generally good fit, and residual plots showed only minor deviations in one or two quantiles. This is not a major concern, particularly given our reasonably sample size.
